# β_3_-adrenoceptors inhibit stimulated norepinephrine release in spontaneously hypertensive rats

**DOI:** 10.3389/fphys.2014.00499

**Published:** 2014-12-19

**Authors:** Torill Berg

**Affiliations:** Department of Physiology, Institute of Basic Medical Sciences, University of OsloOslo, Norway

**Keywords:** β_3_-adrenoceptors, putative β_4_-adrenoceptors, norepinephrine release, epinephrine secretion, hypertension

## Abstract

Here, the influence of β_3_-adrenoceptors on catecholamine release in normotensive and spontaneously hypertensive rats was analyzed. Blood pressure was recorded through a femoral artery catheter, and cardiac output by ascending aorta flow. Time from onset of flow to maximum rise in flow indicated inotropy. Total peripheral vascular resistance (TPR) was calculated. Norepinephrine release was stimulated with tyramine, which allowed presynaptic release-control to be reflected as changes in the plasma norepinephrine concentration. β_3_-adrenoceptor agonist (BRL37344) reduced baseline vascular resistance, the tyramine-stimulated norepinephrine overflow and the positive inotropic response to tyramine in hypertensive but not normotensive rats. β_3_-adrenoceptor antagonist (SR59230A) reduced tyramine-stimulated norepinephrine release in both strains and the secretion of epinephrine in hypertensive rats. SR59230A reduced tyramine-induced tachycardia in normotensive rats, and prevented down-regulation of the tyramine-induced rise in resistance in hypertensive rats. It was concluded that the contradicting results obtained by agonist vs. antagonist, could be explained by their interaction with two different β-adrenoceptors: The BRL37344-dependent inhibition of stimulated norepinephrine release and positive inotropic response to tyramine was compatible with stimulation of β_3_-adrenoceptor coupling to inhibitory G-protein. This was observed only in hypertensive rats during stimulated, high levels of circulating catecholamines. The effect of BRL37344 on baseline vascular resistance was compatible with activation of β_3_-adrenoceptor coupling to endothelial nitric oxide synthase. The inhibitory effect of SR59230A on tyramine-stimulated norepinephrine release in both strains, the increased TPR-response to tyramine in hypertensive rats and tachycardia in normotensive rats may result from inhibition of the low-affinity-state β_1_-adrenoceptor, also known as the putative β_4_-adrenoceptor.

## Introduction

The release of norepinephrine from vesicles in peripheral sympathetic nerve terminals is modulated by presynaptic receptors, which either inhibit or stimulate release (Westfall, 1977; Starke et al., 1989). Of the three β-adrenoceptor (AR) subtypes, i.e., β_1_-, β_2_- and β_3_AR, the β_2_-subtype has been recognized as the main βAR which stimulates norepinephrine release (Stjarne and Brundin, 1976; Westfall et al., 1979; Nedergaard and Abrahamsen, 1990). However, during tyramine-induced norepinephrine release, presynaptic β_1_AR was recently found to be equally efficient as the β_2_AR in stimulating the release of norepinephrine (Berg, 2014). However, their effect was not additive, since blocking both was not more efficient than blocking one or the other. Little is known about the role of the β_3_AR in modulating norepinephrine release, although an increase in norepinephrine transmission, probably in the locus coeruleus, has been observed after central stimulation with the β_3_AR agonist SR58611A (Claustre et al., 2008). However, unlike β_1_- and β_2_AR, the β_3_AR may couple not only to stimulatory G-protein (G_s_) but also to inhibitory G-protein (G_i_) (Gauthier et al., 1996). Through this, the β_3_AR may activate nitric oxide (NO) synthase (NOS), most likely endothelial NOS (eNOS), and produce a negative inotropic effect in cardiomyocytes (Gauthier et al., 1998). In the vasculature, β_3_AR, through coupling to G_s_, may induce vascular smooth muscle cell (VSMC) relaxation directly, or indirectly through endothelial eNOS activation (for review, see Rozec and Gauthier, 2006). If the β_3_AR will inhibit or facilitate norepinephrine release from peripheral sympathetic nerve terminals is not known. In cultured human adrenal chromaffin cells, β_2_AR and β_3_AR, but not β_1_AR, were found to stimulate release of both norepinephrine and epinephrine (Cortez et al., 2012). Similar studies have not been done on the rat adrenal gland or *in vivo*.

Little is known about the role of the β_3_AR in hypertension. A missense mutation in the β_3_AR gene was associated with hypertension (Tonolo et al., 1999; Ringel et al., 2000; Hao et al., 2004) and other features of the metabolic syndrome such as insulin resistance and in some populations, overweight/obesity and dyslipidemia (Widen et al., 1995; Arner and Hoffstedt, 1999; Thomas et al., 2000). This mutation was also associated with an increased sensitivity to the pressor effect of exogenous norepinephrine (Melis et al., 2002), similar to the augmented rise in total peripheral vascular resistance (TPR) during stimulated norepinephrine release in the presence of β_3_AR antagonist (Berg et al., 2010). The β_3_AR is more resistant to catecholamine-induced desensitization than β_1/2_AR, and is preserved and present in a higher density compared to the β_1_AR in SHR (Mallem et al., 2004; Rouget et al., 2004). This difference may contribute to the altered presynaptic control of catecholamine release known to be present in spontaneously hypertensive rats (SHR) (Berg, 2013; Berg and Jensen, 2013).

**Figure 1:**
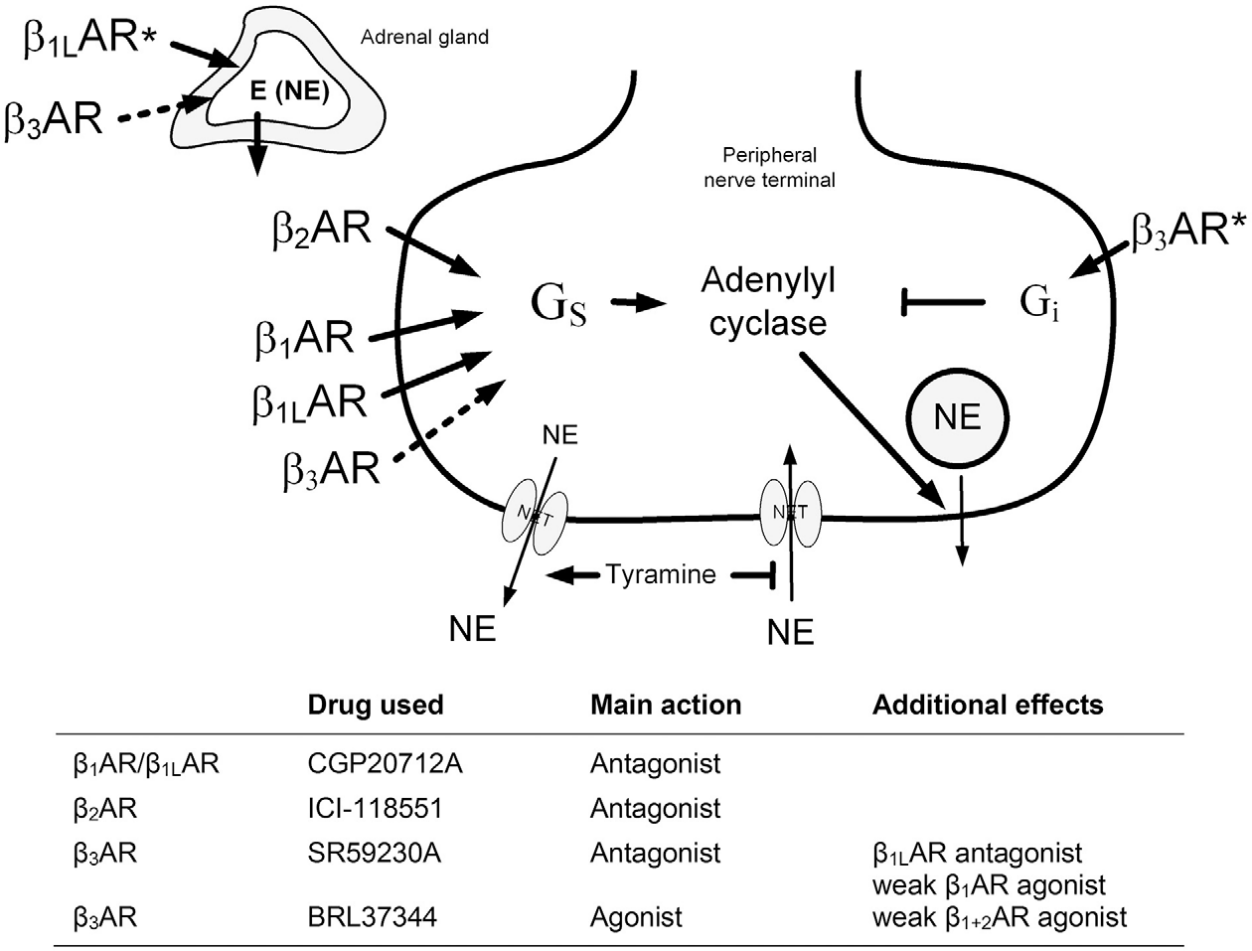
**An overview of the experimental design to test the role of β_3_AR in catecholamine release**. The presynaptic βAR will be activated by the released norepinephrine or by circulating epinephrine from the adrenals. Dotted arrows; tested, but not verified hypothesis. ^*^; effect observed in SHR only in response to β_3_AR antagonist (adrenal) and antagonist (neuronal). NE, norepinephrine; E, epinephrine; NET, norepinephrine re-uptake transporter.

It was therefore hypothesized that β_3_AR are located presynaptically on peripheral sympathetic nerve endings and adrenal chromaffin cells, where they may either lower or facilitate release, and that their function may be more prominent in hypertension. The aim of the present study was therefore to decipher the role of β_3_AR in the control of catecholamine release in SHR and their normotensive controls (WKY). Presynaptic control of norepinephrine release was studied by the use of tyramine, which selectively activates the release of norepinephrine from peripheral sympathetic nerve terminals by stimulating reverse transport through the norepinephrine re-uptake transporter (NET). When NET is blocked (Berg et al., 2012), or engaged in release in the presence of tyramine (Berg and Jensen, 2013), re-uptake is prevented, and the impact of presynaptic control of vesicular release is reflected as differences in overflow to plasma (Berg, 2013, 2014) (Figure 1). The presynaptic receptors are activated by the released norepinephrine and other agonists present in their vicinity. The release of norepinephrine is therefore not directly dependant on the sympathetic tone, which will be influenced by factors such anesthesia, ventilation and rat strain, but activated pharmacologically in the nerve terminal. The surgical trauma was responsible for some secretion of epinephrine, also subjected to presynaptic control (Berg et al., 2012). The present study is the first to analyse *in vivo* the role of β_3_AR in catecholamine release in WKY and SHR, and, in the same animal, their impact on inotropy, heart rate (HR) and TPR.

## Materials and methods

### Experimental procedure

All experiments were approved by The Norwegian Animal Research Authority (NARA) (approval number 10.2914), and conducted in accordance with the Directive 2010/63/EU of the European Parliament. Fifty-eight male, 12–14 weeks old SHR (Okamoto, SHR/NHsd strain, 281 ± 3 g body weight) and 55 age-matched WKY (Wistar Kyoto, 282 ± 3 g body weight) on conventional rat chow diet (0.7% NaCl) were included in the study. As previously described (Berg et al., 2010), the rats were anesthetized with sodium pentobarbital (65–75 mg/kg, IP). The level of surgical anesthesia was tested by non-responsiveness to pinching between the toes. When satisfactory anesthesia was established, it remained throughout the experiment without further supply. The rats were instrumented with a heparinized catheter in the femoral artery to record systolic (SBP) and diastolic (DBP) blood pressure (BP), and a flow probe on the ascending aorta to measure cardiac output (CO) and HR. Mean arterial BP [MBP = (SBP–DBP)/3 + DBP] and TPR (MBP/CO) were calculated. T_F_ (time from onset of ascending aorta flow to maximum rise in flow, derived from high resolution aorta flow data, i.e., 5000 points during a 2-s collection-period, stored when pressing a specified key on the computer) was used to indicate changes in inotropy (Berg et al., 2010). A negative change in T_F_ indicated a positive inotropic response, and *vice versa*. The rats were kept on a positive-pressure respirator and ventilated with air throughout the experiment. Body temperature was maintained by external heating, guided by a thermo sensor inserted inguinally into the abdominal cavity. Removal of the adrenal glands (AdrX) was done through bilateral flank incisions at the start of the surgical procedure, i.e., 30 min before injecting the first drug. At completion of the experiment, the animals were sacrificed by an IV injection of about 35 mg pentobarbital.

### Experimental protocols

All drugs were dissolved in phosphate-buffered saline (PBS; 0.01 M Na-phosphate, pH 7.4, 0.14 M NaCl) and administered through a catheter in the femoral vein. After a control period of about 10 min, control rats were pre-treated with PBS (0.6 ml/kg, bolus injection), followed 10 min later by a 15 min infusion with tyramine (1.26 μmol/kg/min) to stimulate norepinephrine release (Berg et al., 2010, 2012). In time control rats, tyramine was replaced by an infusion with PBS. To test the influence of β_3_AR on release control, the rats were pre-treated with the β_3_AR agonist BRL37344 ((±)-(R^*^, R^*^)-[4-[2-[[2-(3-Chlorophenyl)-2-hydroxyethyl]amino]propyl]phenoxy]-acetic acid sodium hydrate) infused at a rate of 1 nmol/kg/min (Malinowska and Schlicker, 1997) for 10 min, and subsequently combined with tyramine throughout the tyramine infusion-period. Alternatively, rats were pre-treated with the β_3_AR antagonist SR59230A (13.8 μmol/kg, 0.6 ml/kg, bolus injection) 5 min before administration of tyramine, as previously described (Berg et al., 2010). These experiments were run in not-AdrX and AdrX rats to study the influence of circulating epinephrine on β_3_AR activity, and if the effect of β_3_AR agonist and antagonist influenced norepinephrine release indirectly by altering adrenal epinephrine secretion. To study the effect of presynaptic β_3_AR on norepinephrine release in the absence of β_1+2_AR stimulation of release and without interference from the adrenals, AdrX WKY and SHR were pre-treated with the β_1_AR antagonist CGP20712A (11 μmol/kg, 0.6 ml/kg, bolus injection) followed 10 min later by the β_2_AR antagonist ICI-118551 (1 μmol/kg initial dose, subsequently infused with 0.3 μmol/kg/min for 10 min), alone or with SR59230A injected 5 min into the infusion of ICI-118551 (Berg et al., 2010). Tyramine was subsequently infused combined with ICI-118551. An overview of drug actions and hypotheses tested are given in Figure 1. The rats in the antagonist and control groups were in part the same as in a previous study (Berg et al., 2010), where the plasma concentration of catecholamines were not measured, except in the AdrX CGP20712A + ICI-118551 + tyramine SHR group. Additional rats were included to overlap in time with the BRL37344-groups, and to have sufficient numbers of plasma when remaining plasma from old controls and the CGP20712A + ICI-118551-treated AdrX SHR group was not sufficient to repeat the measurement of catecholamines with our present assay, which differed from that previously employed. The cardiovascular response in these additional rats corresponded to that observed before (Berg et al., 2010). Previously published cardiovascular data will therefore be only shortly summarized, to be included in the discussion of the interpretation of the data, in light of the present new information, i.e., the plasma catecholamine concentrations after β_3_AR agonist and antagonist, as well as the cardiovascular response to agonist. The number of rats included in each group (Table 1) was based on sample power calculations using previous data from similar or related experiments.

**Table 1:** **The plasma concentration of norepinephrine and epinephrine**.

	**WKY**			**SHR**		
	**N**	**Norepinephrine (nM)**	**Epinephrine (nM)**	**N**	**Norepinephrine (nM)**	**Epinephrine (nM)**
PBS + PBS (not-AdrX | time control)	6	0.7±0.2	6.9±1.2	6	1.3±0.2	10.9±1.9
PBS + tyramine (not-AdrX control)	9	21.5±1.2^*^	3.9±1.2	9	27.2±1.3^*^^†^	5.8±0.9
BRL37344 + tyramine (not-AdrX)	7	26.1±3.5	4.9±2.3	6	21.8±1.2^‡^	5.4±1.1
SR59230A + tyramine (not-AdrX)	10	14.9±2.1^‡^	1.8±0.6	9	21.2±1.6^‡^	2.7±1.0^‡^
AdrX + PBS + PBS (AdrX time control)	6	0.2±0.1	0.0±0.0	8	3.7±1.3	0.3±0.2
AdrX + PBS + tyramine (AdrX control)	6	21.7±2.4^*^	0.7±0.7^⊣^	6	33.2±4.0^*^^†^	0.1±0.1^⊣^
AdrX + BRL37344 + tyramine	8	17.3±1.9^⊣^	0.0±0.0^⊣^	6	34.6±5.7^⊣^	0.0±0.0^⊣^
AdrX + SR59230A + tyramine	8	22.6±2.9^⊣^	0.4±0.4^⊣^	7	33.2±5.1^⊣^	0.4±0.3^⊣^
AdrX + CGP20712A + ICI-11855 + tyramine	7	16.7±2.4	0.6±0.4	7	37.7±5.3	0.2±0.2
AdrX + CGP20712A + ICI-11855 + SR59230A + tyramine	6	12.8±1.3^‡^	0.9±0.9	6	25.6±6.5	0.5±0.5

Significant differences between the time controls and corresponding tyramine control groups (^*^), between the SHR and WKY PBS + tyramine groups in not-AdrX and AdrX rats (^†^ after SHR values), between corresponding PBS + tyramine and agonist/antagonist + tyramine groups (^‡^), and between corresponding not-AdrX and AdrX groups (^⊣^), were detected as indicated. Significant differences between the AdrX + CGP20712A + ICI-11855 + tyramine and AdrX + CGP20712A + ICI-11855 + SR59230A + tyramine groups were not detected. N, number of rats per group.

*, †, ‡, ⊣ *— P ≤ 0.05*.

### Measurement of plasma catecholamines

At the end of the tyramine infusion, without discontinuing the infusion, 1.5 ml blood was collected by free flow from the femoral artery catheter, into tubes containing 40 μl 0.2 M glutathione, 0.2 M ethylene glycol-bis(2-aminoethylether)-N,N,N′,N′-tetraacetic acid (EGTA) (4°C). Plasma was stored at −80°C until catecholamine concentrations were determined using 400 μl plasma and the 5000 Reagent kit for HPLC analysis of Catecholamines in plasma from Chromsystems GmbH, Munich, Germany, as described by the manufacturer. The samples were run on a Shimadzu monoamines analyzer system, using an isocratic flow rate of 0.8 ml/min, and an electrochemical detector (Decade II) and a SenCell electrochemical flow cell (Antec Leyden, Zoeterwoude, The Netherlands).

### Drugs

SR59230A was from Santa Cruz Biotechnology, Heidelberg, Germany, and ICI-118551 from ICI-Pharma, Cheshire, UK. BRL37344 and tyramine were from Sigma Chemical Co., St. Louis, MO, USA.

### Statistical analyses

The results are presented as mean values ± s.e.m. The plasma catecholamine concentrations were evaluated by overall tests (One-Way ANOVA). When the presence of significant group differences was indicated, these were located by two-sample Student's *t*-tests for parametric data, and by Kruskal-Wallis tests for non-parametric data. *P* ≤ 0.05 was considered significant.

The cardiovascular data were averaged every min. The cardiovascular response to pre-treatment and baselines prior to tyramine were evaluated by One-Way ANOVA, including all groups within each strain. When the presence of group differences was indicated, these were subsequently located by two-sample Student's *t*-tests and Kruskal-Wallis tests for parametric and non-parametric results, respectively. The tyramine response-curves were analyzed using Repeated Measures Analyses of Variance and Covariance, first as over-all tests including all groups within each strain, and subsequently between groups and for each group separately. Significant responses (one-sample Student's *t*-tests) and groups differences (two-sample Student's *t*- or Kruskall-Wallis tests) were subsequently located at specific times, i.e., at the initial peak-pressure response (about 3 min) and/or after 15 min. For each step, testing proceeded only when the presence of significant responses, differences and/or interactions was indicated, and the *P*-value was for all tests and each step adjusted according to Bonferroni.

## Results

### Effect of β_3_AR-agonist and -antagonist on the plasma catecholamine concentrations (Table 1)

Tyramine clearly increased the plasma concentration of norepinephrine in both not-AdrX and AdrX rats of both strains (*P* < 0.001 compared to the time controls infused with PBS instead of tyramine), but did not significantly influence the epinephrine concentration. At the end of the tyramine-infusion period, the plasma concentration of norepinephrine was higher in SHR than in WKY in not-AdrX as well as AdrX rats (*P* = 0.005 and 0.037, respectively). The concentration of norepinephrine in AdrX rats was not different from that in the not-AdrX controls (*P* = NS), showing that the tyramine-stimulated overflow of norepinephrine involved sympathetic nerves rather than the adrenals. The plasma concentration of epinephrine was almost totally eliminated in AdrX rats (*P* = 0.029 and 0.001 compared to not-AdrX WKY and SHR, respectively).

The β_3_AR-agonist BRL37344 did not alter the tyramine-induced norepinephrine overflow to plasma in WKY (*P* = NS) but reduced overflow in SHR (*P* = 0.011), suggesting β_3_AR to have a negative influence on release in this strain. In contrast to this result, the β_3_AR-antagonist SR59230A was observed to lower norepinephrine overflow, and did so in both strains (*P* = 0.006 and 0.032 in WKY and SHR, respectively). SR59230A and BRL37344 had no significant effect on norepinephrine overflow in AdrX WKY and SHR.

In AdrX rats, pre-treatment with β_1_- and β_2_AR antagonist, i.e., CGP20712A + ICI-118551, followed by SR59230A reduced the tyramine-stimulated norepinephrine overflow (*P* = 0.018 compared to the AdrX WKY controls), whereas the almost same reduction following CGP20712A + ICI-118551 alone was not statistically significant. The reduction seen in CGP20712A + ICI-118551 + SR59230A-pre-treated AdrX SHR was not statistically significant (*P* = NS compared to the AdrX SHR controls).

BRL37344 had no effect on the plasma epinephrine concentration in either strain (*P* = NS). However, SR59230A reduced the plasma epinephrine concentration in both strains, although the difference was statistically significant in SHR only (*P* = 0.034).

### Effect of β_3_AR-agonist and -antagonist on cardiovascular baselines

BRL37344 reduced MBP (−11 ± 2 compared to the −1 ± 2 mm Hg following the injection of PBS in the controls, *P* = 0.005) in WKY, increased CO in both strains (9 ± 2 and 4 ± 1 ml/min in WKY and SHR, respectively, compared to 3 ± 0 and 1 ± 1 ml/min in the controls) and in AdrX WKY (7 ± 1 compared to 3 ± 1 ml/min in the controls) (*P* ≤ 0.006). The effect of BRL37344 on TPR baseline was not significant in WKY or SHR (*P* = NS). However, BRL37344 reduced baseline TPR in the AdrX WKY (−0.9 ± 0.1 mm Hg/ml/min compared to −0.4 ± 0.1 mm Hg/ml/min after PBS) and AdrX SHR (−1.2 ± 0.4 and −0.4 ± 0.4 mm Hg/ml/min, respectively). The resulting TPR after pre-treatment, i.e., prior to tyramine, was 2.4 ± 0.3 and 1.9 ± 0.1 mm Hg/ml/min after PBS and BRL37344, respectively in the WKY controls, 2.1 ± 0.1 and 1.9 ± 0.2 mm Hg/ml/min, respectively, in AdrX WKY, and 5.0 ± 0.3, 4.8 ± 0.3, 6.6 ± 0.9 (*P* = NS compared to the control group) and 3.6 ± 0.8 (*P* = 0.034) mm Hg/ml/min in the corresponding SHR groups. BRL37344 reduced T_F_ (i.e., increased inotropy) in SHR (Δ*T*_F_ = −6 ± 4 compared to 4 ± 3% in the controls) and in AdrX SHR (Δ*T*_F_ = −7 ± 3 compared to 2 ± 5% in the controls) (*P* ≤ 0.01). As previously described (Berg et al., 2010), the antagonist SR59230A increased baseline MBP and reduced T_F_ (i.e., indicating an increase in inotropy) in WKY (*P* ≤ 0.004), and increased MBP and CO and reduced T_F_ in AdrX WKY (*P* ≤ 0.018) (data not shown). A change in baseline T_F_ was not observed in AdrX WKY when SR59230A was given after β_1_- and β_2_AR blockade with CGP20712A and ICI-118551 (−8 ± 2% compared to 1 ± 2% in the control group, *P* = 0.041). SR59230A had no effect on TPR baseline in not-AdrX or AdrX WKY or any of the cardiovascular baselines in not-AdrX or AdrX SHR. TPR after SR59230A was 3.3 ± 0.3 and 3.3 ± 0.4 mm Hg/ml/min in WKY and AdrX WKY, respectively, and 5.4 ± 0.4 and 7.3 ± 0.8 mm Hg/ml/min in the corresponding SHR groups.

### Effect of β_3_AR-agonist and -antagonist on the cardiovascular response to tyramine

**Figure 2:**
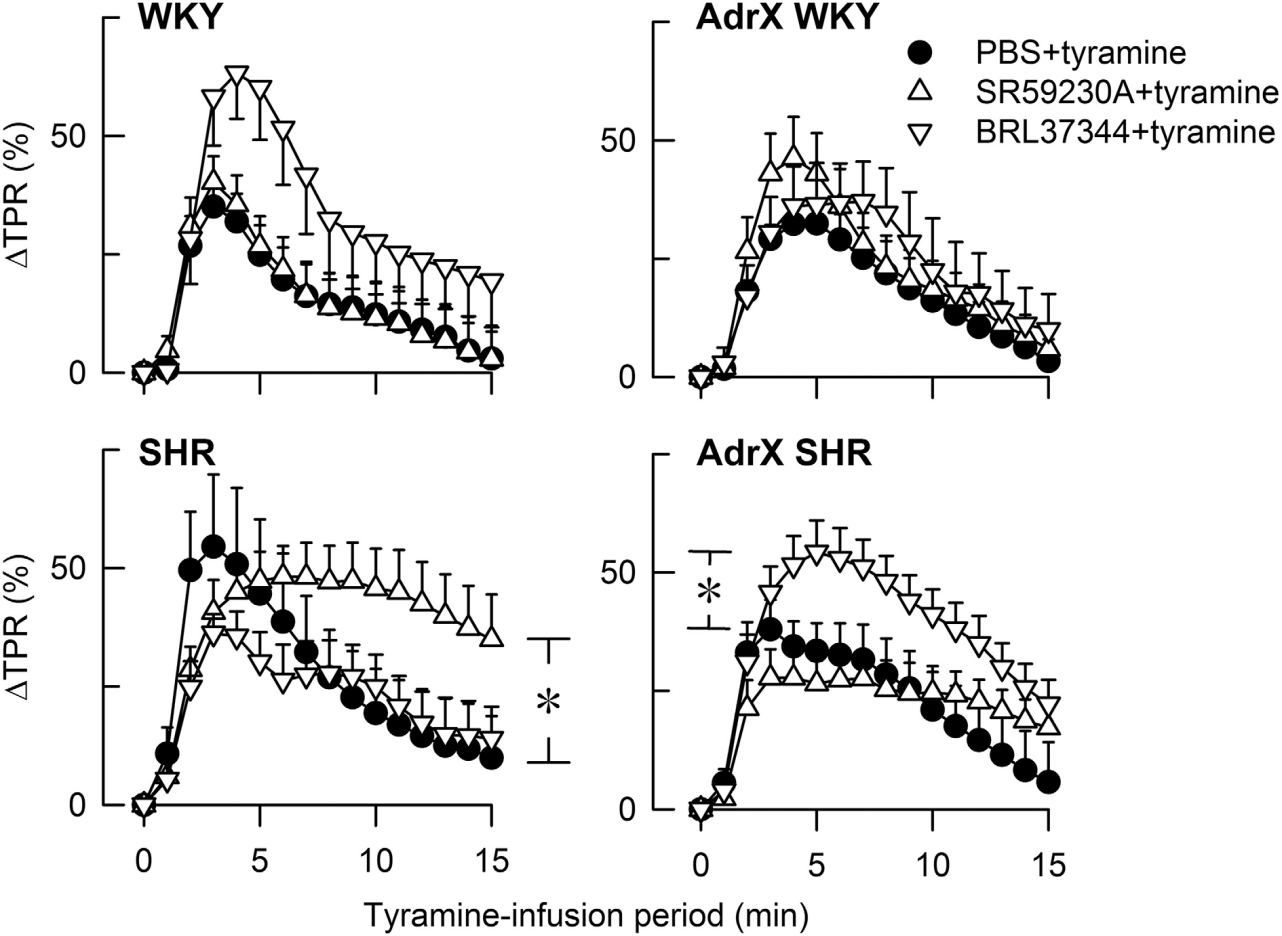
**The TPR-response to tyramine in WKY and SHR, without and after acute AdrX**. The rats were pre-treated with the β_3_AR antagonist SR59230A or agonist BRL37344 as indicated by symbol legends. The TPR-results in the SR59230A-treated groups are from Berg et al. (2010). ^*^P ≤ 0.025 after curve evaluation (please see Methods).

BRL37344 reduced the tyramine-induced fall in T_F_ in not-AdrX SHR (−23 ± 2 compared to −36 ± 2% in the controls, *P* = 0.018), demonstrating a counter-action of the positive inotropic response to tyramine. BRL37344 had no effect on the tyramine-induced, sustained tachycardia in none of the groups (data not shown). The same pattern was seen regardless of expressing the change in bpm or in percentage of HR baseline. As previously described (Berg et al., 2010), SR59230A clearly reduced the tachycardia in WKY and AdrX WKY but had no effect in SHR or AdrX SHR. The rise in TPR in response to tyramine was augmented by BRL37344 throughout the tyramine infusion-period in AdrX SHR when expressed in percentage of baseline (Figure 2), but not when expressed in mm Hg/ml/min, and may therefore result from the particularly low TPR baseline after AdrX in this group. The down-regulation of the TPR-response to tyramine observed during the late part of the infusion-period was eliminated by SR59230A in not-AdrX SHR (Figure 2).

## Discussion

The main result in the present study was that the β_3_AR agonist BRL37344 reduced tyramine-stimulated norepinephrine overflow to plasma in SHR but not in WKY. BRL37344 also reduced the positive inotropic response to tyramine. Most effects of the antagonist SR59230A were likely to be due to inhibition of low-affinity state β_1_AR (β_1L_AR), i.e., a reduced tyramine-stimulated norepinephrine release in both strains, a reduced secretion of epinephrine in SHR and a reduced tyramine-induced tachycardia in WKY. BRL37344 reduced TPR baseline in SHR, and SR59230A prevented the down-regulation of the TPR-response to tyramine, compatible with effects involving β_3_AR-eNOS- or β_1L/1/2_AR-mediated vasodilatation. The results and deductions are summarized in Table 2.

**Table 2:** **Effects of the agonist BRL37344 and the antagonist SR59230A, and possible interpretations of the results**.

		**WKY**	**AdrX WKY**	**SHR**	**AdrX SHR**	**Possible mechanisms responsible for the results**
**BRL37344**						
Baselines:	T_F_	~	~	↓	↓	Pos. inotropy: ↑ β_1_/β_2_AR
	TPR	~	↓	~	↓	^a^Vasorelaxation: ↑ β_3_AR-eNOS or ↑ VSMC β_1/2_AR-G_s_
Tyramine:	T_F_	~	~	↑	~	Neg. inotropy: ↑ β_3_AR-G_i_ or due to ↓NE release
	TPR	~	~	~	(↑)	^a^Vasoconstriction: Due to a low baseline?
	Plasma NE	~	~	↓	~	Inhibition of release: ↑ β_3_AR-G_i_
	Plasma EPI	~	~	~	~	No effect on adrenal epinephrine release
**SR59230A**						
Baselines:	T_F_	↓	↓	~	~	Pos. inotropy, absent after CGP20712A+ICI-11855: ↑ β_1_AR-G_s_
	TPR	~	~	~	~	No effect
Tyramine:	T_F_	~	~	~	~	No effect
	HR	↓	↓	~	~	Neg. chronotropy: ↓ β_1L_AR-G_s_ or due to ↓NE release
	TPR	~	~	↑	~	↓ βAR vasorelaxation: ↓ β_3_AR-eNOS and/or ↓ β_1L_AR-G_s_
	Plasma NE	↓	~	↓	~	Inhibition of release: ↓ β_1L_AR-G_s_
	Plasma EPI	~	~	↓	~	Inhibition of release: ↓ β_1L_AR-G_s_

a *In the absence of epinephrine-activated β_2_AR-mediated vasodilatation. NE, norepinephrine. EPI, epinephrine, secretion activated by the surgical trauma, not by tyramine. ~, unchanged parameter. Pos., positive. Neg., negative*.

### The effect of β_3_AR-agonist and -antagonist on stimulated norepinephrine release

The influence of β_3_AR on catecholamine release was studied during tyramine-stimulated norepinephrine release since βAR antagonist (propranolol) did not significantly alter the low plasma catecholamine concentrations in unstimulated rats (T. Berg, unpublished observations). As previously published (Berg, 2014), the tyramine-stimulated norepinephrine overflow was not different in AdrX rats of either strain, showing that the released norepinephrine originated from peripheral sympathetic nerves rather than the adrenals. The agonist BRL37344 had no effect on the tyramine-stimulated norepinephrine overflow in WKY, but reduced overflow in SHR. This was not explained by the weak β_1_- and β_2_AR agonistic effect of BRL37344 (Dolan et al., 1994), since such activity would be expected to enhance release. BRL37344 may therefore inhibit norepinephrine release by stimulating β_3_AR-coupling to G_i_, similar to that described for the β_3_AR-mediated inhibition of cardiomyocyte contraction (Gauthier et al., 1996). These inhibitory β_3_AR were likely to be located presynaptically on peripheral sympathetic nerve endings, similar to that described for the facilitating β_1_AR and β_2_AR (Westfall, 1977; Starke et al., 1989; Berg, 2014).

Similar to that previously described for both β_1_- and β_2_AR antagonists (Berg et al., 2010; Berg, 2014), also the β_3_AR antagonist SR59230A reduced the tyramine-induced norepinephrine overflow in WKY and SHR. Any β_1_AR agonistic effect of SR59230A (Malinowska and Schlicker, 1997) would be expected to increase norepinephrine overflow. Thus, opposite of what was expected from the effect of BRL37344, and under the same conditions, SR59230A inhibited a release-stimulating mechanism. In addition, unlike the β_3_AR-G_i_-signaling suggested by BRL37344, this stimulating mechanism was present in both strains. However, SR59230A has been shown *in vivo* to inhibit an atypical, putative, G_s_-coupled, cardio-stimulating βAR with equal efficacy as it inhibited β_3_AR-mediated thermogenesis, whereas the same receptor was only marginally stimulated by BRL37344 (Malinowska and Schlicker, 1997). This putative βAR has now been recognized to represent β_1L_AR (Granneman, 2001; Kaumann et al., 2001). Since norepinephrine release has been shown to be reduced by β_1_AR antagonists such as atenolol and metoprolol and also the β_1L_AR antagonist CGP20712A (Berg, 2014; Berg et al., 2010), it seemed reasonable to conclude that the reduced norepinephrine overflow in SR59230A-treated rats resulted from inhibition of presynaptic β_1L_AR.

SR59230A and BRL37344 did not significantly influence the tyramine-stimulated norepinephrine overflow to plasma in AdrX rats of either strain. This observation may indicate that circulating epinephrine was in fact the agonist responsible for the β_1L_AR-activity, at least in WKY, and that the effect of β_3_AR agonist/antagonist on norepinephrine release was indirect and due to an effect on the secretion of epinephrine. However, as discussed below, a significant change in the plasma epinephrine concentration was observed only in the SR59230A-pre-treated SHR. β_1 + 2_AR inhibition, i.e., CGP20712A and ICI-118551 combined, reduced overflow in AdrX WKY, although the difference was not statistically significant, and additional pre-treatment with SR59230A resulted in a similar, significant reduction in the plasma norepinephrine concentration. An additive effect of βAR antagonist directed against more than one subtype may not have been expected since the reduction following inhibition of β_1_AR and β_2_AR separately was not different from that after inhibition of both (Berg, 2014). However, pre-treatment with CGP20712A+ICI-118551, alone or combined with SR59230A, did not reduce norepinephrine overflow in AdrX SHR. Thus, AdrX altered the ability of not only the β_3_AR, but also that of β_1 + 2_AR, to stimulate norepinephrine release. This change may be due to that AdrX altered the balance between the βAR and other mechanism(s) influencing norepinephrine release, for instance α_2_AR-mediated inhibition of release. This possibility is presently under investigation.

### The effect of β_3_AR-agonist and -antagonist on epinephrine release

The plasma concentration at the end of the experiment in time controls was higher than that in plasma from anesthetized rats not subjected to surgery other than femoral artery catheterization (0.1 ± 0.1 nM in both WKY and SHR compared to 6–10 nM in the time controls, *P* < 0.001) (Berg et al., 2012). It was therefore concluded that the adrenal secretion of epinephrine in this experimental model was activated by the surgical trauma. Also this release was sensitive to α_2_AR auto-inhibition (Berg, 2013; Berg and Jensen, 2013). Pre-treatment with SR59230A, but not BRL37344, reduced the plasma concentration of epinephrine, with a statistically significant difference detected only in SHR. The secretion of epinephrine in SHR therefore appeared to be enhanced by the β_1L_AR. SR59230A, like the β_2_AR antagonist ICI-118551, also reduced basal and stimulated catecholamine release in human adrenal, isolated chromaffin cells, indicating a β_2_- and β_3_AR-mediated stimulation of release (Cortez et al., 2012). Unless the β_3_AR influence on epinephrine and norepinephrine release in the rat differed by coupling to G_s_ and G_i_, respectively, it may be considered that SR59230A may inhibit the β_1L_AR also in human chromaffin cells.

### The effect of β_3_AR-agonist and -antagonist on cardiovascular baselines

BRL37344 induced a decrease in baseline T_F_ in SHR but not WKY, indicating a positive inotropic effect. The same response was observed also in AdrX SHR. Without stimulation of norepinephrine release, overflow to plasma in anesthetized rats was very low, even in the presence of desipramine, an inhibitor of synaptic norepinephrine re-uptake (Berg et al., 2012). If β_3_AR-activation depended on a high catecholamine concentration and, from that, a catecholamine-induced receptor rearrangement, the β_3_AR may not be present in sufficient quantity in the basal condition to mediate a negative inotropic response. The positive inotropy may therefore without stimulation of norepinephrine release result from the weak β_1 + 2_AR agonistic effect of BRL37344 (Dolan et al., 1994). BRL37344 in addition induced a fall in TPR baseline in AdrX rats of both strains. Thus, in the absence of the β_2_AR-mediated vasodilatory component, BRL37344 induced vasodilatation, either through a VSMC β_1/2_AR-G_s_-cAMP-dependent mechanism or, perhaps more likely, through endothelial β_3_AR-eNOS activation (Mallem et al., 2005).

SR59230A reduced baseline T_F_ in both not-AdrX and AdrX WKY, but not in SHR, demonstrating an increase in inotropy, suggesting the presence of a β_3_AR-G_i_-mediated, negative inotropic component in the unstimulated WKY (Berg et al., 2010). However, SR59230A has been shown to have some β_1_AR agonistic effect (Malinowska and Schlicker, 1997), which will be expected to enhance inotropy and thus lower T_F_. This explanation was supported by the fact that SR59230A did not lower baseline T_F_ after prior administration of β_1 + 2_AR antagonists in AdrX WKY. Thus, what appeared to indicate a SR59230A-induced inhibition of a β_3_AR-G_i_-mediated negative inotropy, as we previously concluded (Berg et al., 2010), may just as well be due to β_1_AR activation.

### The effect of β_3_AR-agonist and -antagonist on the cardiovascular response to stimulated norepinephrine release

When the release of large amounts of norepinephrine was stimulated by tyramine, the results differed from that observed in the basal condition. BRL37344 increased T_F_ in tyramine-stimulated SHR but not in AdrX SHR or WKY, indicating that BRL37344 in SHR hampered the positive inotropic effect of tyramine. Thus, selective β_3_AR stimulation with agonist in the presence of high concentrations of norepinephrine and circulating epinephrine appeared to evoke coupling to G_i_, possibly functioning as a “safety valve” against excessive stimulation. This may be due to a direct effect on the cardiomyocytes, but may also be explained by the parallel reduction in norepinephrine release, which also was observed only in the not-AdrX SHR group. A reduction in norepinephrine release may also explain the previously documented inhibitory effect of SR59220A on the tyramine-induced tachycardia in WKY (Berg et al., 2010). The SR59230A-dependant inhibition of the down-regulation of the late TPR-response to tyramine in not-AdrX SHR was compatible with an up-regulated β_3_AR expression in SHR, and a SR59230A-dependant inhibition of β_3_AR-eNOS induced vasodilatation. However, it cannot be excluded that the latter two effects of SR59230A may be explained by inhibition of β_IL_AR-G_s_-signaling, since also the β_1_AR antagonist CGP20712A reduced the tyramine-induced tachycardia and prevented the down-regulation of the late TPR-response (Berg et al., 2010).

## Conclusions

From the present results it may be deduced that effects explained by a β_3_AR-G_i_-signaling, i.e., inhibition of norepinephrine release and negative inotropy, was only observed after stimulation with the agonist BRL37344, and then only in SHR during tyramine-stimulated release of norepinephrine. In the absence of selective, exogenous β_3_AR agonist, the response to tyramine-stimulated norepinephrine release predominantly involved β_IL_AR, such as stimulation of norepinephrine release in both strains, secretion of epinephrine from the adrenal glands in SHR and norepinephrine-induced tachycardia in WKY. These responses were inhibited by SR59230A, which antagonizes β_1L_AR equally well as β_3_AR. In situations such as heart failure, acute myocardial infarction and arrhythmia, where the release of norepinephrine is greatly increased, BRL37344 and SR59230A may both provide relief by reducing norepinephrine release.

## Funding

This work was supported by The Norwegian Council on Cardiovascular Diseases and by Anders Jahres Fond.

### Conflict of interest statement

The author declares that the research was conducted in the absence of any commercial or financial relationships that could be construed as a potential conflict of interest.
